# Prognosis of Postoperative Pulmonary Embolism in High Altitude

**DOI:** 10.7759/cureus.46358

**Published:** 2023-10-02

**Authors:** Fadi S Alosaimi, Osama H Al Sayed, Muhanna A Alhusayni, Abdulrahman Alsubaie, Abdullah Ibrahim M Algethami, Mohammad Eid M Mahfouz

**Affiliations:** 1 Medicine, Taif University, Taif, SAU; 2 Surgery, Taif University, Taif, SAU

**Keywords:** saudi arabia, prognosis, high altitude, highlanders, pulmonary embolism

## Abstract

Background: Pulmonary embolism (PE) is a common cause of death and serious disability, with risks that extend beyond the acute phase. Despite advances in diagnosis and treatment, high mortality rates remain a persistent problem.

Aim: The current study aimed to investigate PE prognosis and its determinants among native highlanders in Taif City, Saudi Arabia.

Methods: This is a retrospective study where data was collected from the medical records of native high-altitude PE patients in Taif, Saudi Arabia, from 2017 to 2022.

Results: The study included 154 native high-altitude PE patients with a mean age of 54±19 years. Most were females and nonsmokers (51.3% (n=79) and 89% (n=137), respectively). Of them, 28.6% (n=44) had undergone a previous surgery, and 61.4% (n=27) of these surgeries were within 1-3 weeks before hospital admission. The majority of patients had sub-massive PE (59.1% (n=91)), followed by non-massive PE (24% (n=37)) and massive PE (16.9% (n=26)). After management, 98 (63.6%) patients were improved, and 56 (36.4%) patients were not improved at the time of data collection. In terms of improvement after PE, patients who had undergone a previous surgery were less than those who had not, with no significant difference (56.8% (n=25) and 66.4% (n=73), respectively, p=0.266). All patients with heart rates (HRs) less than 70 bpm improved after PE compared to those with higher HRs (p=0.003). The thrombus location had no statistically significant association with patient outcomes (p=0.058).

Conclusion: This study provides valuable insights into patient outcomes at high altitudes after PE and the prognostic factors influencing these outcomes. It was identified that a low HR was associated with positive outcomes.

## Introduction

A pulmonary embolism (PE) arises when a blood clot originating from another location obstructs the blood flow within the pulmonary artery or its branches. In deep vein thrombosis (DVT), the formation of a blood clot, known as a thrombus, within the deep veins typically occurs in the lower extremities. PE typically results from a thrombus fragment entering the pulmonary circulation, but it can also occur due to the embolization of air, fat, or tumor cells. Venous thromboembolism (VTE) describes a combination of PE and DVT [1].

PE and VTE rank as the third most common causes of cardiovascular mortality [2]. The disease has an incidence of 0.5-1 cases per 1,000 [3]. In Saudi Arabia, the incidence rate of PE is estimated to be 5.5% [4].

Major adverse outcomes associated with untreated PE include recurrent thromboembolism, chronic thromboembolic pulmonary hypertension, post-thrombotic syndrome, and death [5]. Hence, early management is crucial to prevent complications or death. Medical treatment involves anticoagulants to prevent new clots from forming. Thrombolytic drugs may be reserved for life-threatening conditions to dissolve clots due to various side effects. In resistant cases, surgical management may be necessary for a catheter to remove clots or a filter to prevent clot passage from the inferior vena cava to the lungs [6].

As a result, early diagnosis is crucial since acute PE can be fatal if not detected and treated promptly. The diagnostic approach of PE includes clinical and risk assessment, pretest probability assessment, D-dimer testing, and certain imaging such as chest radiograph, computed tomography (CT) scan, and magnetic resonance imaging (MRI) [5].

Nearly 25% of VTE cases are attributed to recent surgical procedures. Over the past 25 years, evidence-based guidelines worldwide have recommended active strategies to prevent VTE in patients who are at risk and undergoing surgery [7]. Major general surgery, including liver, gastric, pancreatic, orthopedic, thoracic, or bowel surgery, leads to a higher risk of developing PE, with a percentage of 0.8%-1.7% [8].

Furthermore, the risk of developing postoperative PE is estimated to be highest within the first five weeks after surgery and varies depending on the type of surgical procedure. International guidelines currently advocate for prophylactic anticoagulation treatment for a duration of up to five weeks following surgery [7].

There are several major risk factors for thromboembolic events, such as recent immobilization, surgery, cardiovascular diseases, recent trauma, older age, prior VTE, malignancy, and indwelling venous catheters. The moderate risk factors include the use of estrogen or hormone replacement therapy, smoking, obesity, pregnancy, and family history of VTE [2].

On the other hand, it was observed that exposure to high altitudes can increase the chance of blood clots during air travel, mountain climbing, or sports [9]. Moreover, Shlim et al. [10] and Dickinson et al. [11] reported that PE is common among climbers.

Although there are scattered case reports and current knowledge of altitude and pulmonary disease, there is no clear association between altitude and the risk of PE or its prognosis. Therefore, our study aims to investigate the prognosis of PE and determine the risk factors that impacted the patients’ outcomes in native highlanders in Taif City. By focusing on this specific population, we aim to contribute to the existing body of knowledge and provide insights into the prognosis of PE in individuals residing at high altitudes. Through our study, we hope to identify the risk factors that influence the outcomes of PE in native highlanders, thereby improving our understanding of this condition and facilitating targeted interventions for better patient care.

## Materials and methods

Study design

A retrospective study was conducted among native high-altitude PE patients in Taif, Saudi Arabia, to investigate the prognosis of PE and factors impacting patient outcomes. The study aimed to explore the relationships between various clinical parameters and prognosis.

Data collection

Data was collected from the medical records of PE patients during the period from 2017 to 2022. The collected variables included demographic information (age and sex), medical history (pregnancy status and chronic diseases), laboratory values (D-dimer, international normalized ratio (INR), red blood cell (RBC) count, white blood cell (WBC) count, hemoglobin, platelet count, blood pH, and cardiac biomarker), clinical measures (blood pressure, systolic blood pressure (SBP), O2 saturation, CO2, and heart rate (HR)), medical conditions (history of deep venous thrombosis (DVT) or PE, cardiac problems, hemodynamic stability, past medical disease, previous surgery, and smoking status), imaging data (location of thrombus), and length of stay (LOS). Moreover, PE types among patients were classified into massive, sub-massive, and non-massive PE. Patient improvement was defined based on the medical records, and patient factors potentially affecting the improvement were investigated.

Statistical methods

Data analysis was performed using Statistical Package for the Social Sciences (SPSS) version 21 (IBM SPSS Statistics, Armonk, NY). Categorical data was described using numbers and percentages, while numerical data was presented using mean and standard deviation (SD) measures, median (interquartile range (IQR)), and range. A normality test was performed for continuous variables. According to the normality test, it was found that the age variable was not normally distributed.

The Monte Carlo test was utilized to explore the relationship between qualitative variables. Since assumptions for Pearson’s chi-square test were violated, the Monte Carlo test was a more appropriate choice. Binary logistic regression was employed to assess the odds between dependent and independent variables, with age as a potential predictor.

A p-value of 0.05 or less was considered statistically significant for all analyses. All statistical tests were two-tailed, ensuring a comprehensive examination of the data.

Ethical considerations

Approval was obtained from the institutional review board of Taif University before conducting any study-related procedures (approval number: HAP-02-T-067). Data was collected anonymously, and data confidentiality was maintained during and after study conduction.

## Results

Baseline characteristics

This study involved 154 native high-altitude PE patients. The mean age of the participants was 54±19 years, and the median (IQR) age was 52 years (31)(range: 18-97 years); 51.3% (n=79) were females, 89% (n=137) were nonsmokers, and 3.9% (n=6) were pregnant. Approximately one-quarter of the patients (23.4%, n=36) have both diabetes and hypertension, and 55.2% (n=85) have heart problems, 12.3% (n=19) had a previous medical history of DVT, and 29.9% (n=46) were using prophylaxis anticoagulants. Furthermore, nearly one-third of the patients (28.6%, n=44) had undergone previous surgery, and 61.4% (n=27) of these surgeries were 1-3 weeks before hospital admission. Full details are identified as shown in Table 1.

**Table 1:** Baseline characteristics of study participants Data has been represented as n, %, mean (SD), median (IQR), and range. n: number, %: percentage, SD: standard deviation, IQR: interquartile range, LV: left ventricle, RV: right ventricle, DVT: deep vein thrombosis, PE: pulmonary embolism, COVID-19: coronavirus disease 2019

Characteristics	n (total=154)	%
Gender		
Female	79	51.3
Male	75	48.7
Age		
Mean±SD	54±19	
Minimum-maximum (range)	18-97 (79)	
Median (IQR)	52 (31)	
Smoking		
No	137	89
Past smoker	2	1.3
Yes	15	9.7
Pregnancy		
Pregnant	6	3.9
Non-pregnant	73	47.4
Chronic disease history		
Diabetes	18	11.7
Hypertension	27	17.5
Hypertension and diabetes	36	23.4
Pulmonary disease with hypertension or diabetes	4	2.6
Other chronic disease	14	9.1
Free history	55	35.7
Heart problems		
Congestive heart failure	5	3.2
Ischemic heart disease	5	3.2
Myocardial infarction	3	1.9
LV dysfunction	5	3.2
RV dysfunction	33	21.4
Other cardiac problems	34	22.1
No cardiac problems	69	44.8
Past medical history		
Cancer	10	6.5
Cardiac disease	17	11
Past COVID-19 infection	12	7.8
Cerebrovascular accident	11	7.1
Hypothyroidism	6	3.9
Pulmonary disease	5	3.2
Others	35	23.7
Free history	58	37.7
History of DVT or PE		
DVT	19	12.3
PE	15	9.7
DVT and PE	12	7.8
None of them	108	70.1
Prophylaxis by anticoagulant		
Yes	46	29.9
No	108	70.1
Previous surgery		
Yes	44	28.6
No	110	71.4
Type of surgery (n=44)		
Abdominal	17	38.6
Cardiac	14	31.8
Orthopedic	13	29.6
Time of surgery (n=44)		
Before 1-3 weeks	27	61.4
Before 3-6 weeks	17	38.6

Clinical characteristics and PE outcome

The majority of patients had sub-massive PE (59.1%, n=91), followed by non-massive PE (24%, n=37) and massive PE (16.9%, n=26). It was subsegmental in the majority of the patients (89%, n=137). Concerning patients’ vital signs, 41.6% (n=64) of the patients had blood pressure above 130/73, and 67.8% (n=103) had a normal HR. Roughly less than half of the patients had oxygen saturation levels below 88.6% (48.7%, n=75) and abnormal CO2 levels (47.4%, n=73), and 37.7% (n=58) of them had abnormal pH levels.

Regarding laboratory tests, 90.9% (n=140) of the participants had normal results for both WBC and platelet counts, 89.6% (n=138) had a normal RBC count, and 84.4% (n=130) had hemoglobin levels above 9.1 g/dL. Almost all patients (99.4, n=153) had medical intervention, with 63.6% (n=98) of patients improved and a LOS of >14 days (51.3%, n=79). Full details are illustrated in Table 2.

**Table 2:** Frequency and percentage of clinical characteristics during hospital stay Data has been represented as n and %. %: percentage, n: number, PE: pulmonary embolism, DVT: deep vein thrombosis, HR: heart rate, SBP: systolic blood pressure, ABG: arterial blood gas, SpO2: saturation of peripheral oxygen, RBC: red blood cell, WBC: white blood cell, LOS: length of stay, ICU: intensive care unit

Characteristics	n (total=154)	%
Initial diagnosis		
Bilateral PE	9	5.8
Massive PE	19	12.3
PE	12	7.8
PE with other conditions	64	41.2
Sub-massive PE	50	32.5
Types of PE		
Massive PE	26	16.9
Sub-massive PE	91	59.1
Non-massive PE	37	24
Location of thrombus		
Pulmonary artery	5	3.2
Segmental	5	3.2
Subsegmental	137	89
Others	7	4.6
Vital signs		
HR		
<71.1 bpm	14	9.2
71.1-105.7 bpm	103	67.8
>105.7 bpm	35	23
Blood pressure		
Above 130/73	64	41.6
Below 115/70	38	24.7
Between 115/70 and 130/73	52	33.8
SBP		
Less than 90	26	16.9
More than 90	128	83.1
Hemodynamic stability		
Yes	62	40.3
No	92	59.7
ABG		
pH		
Abnormal	58	37.7
Normal	96	62.3
CO2		
Abnormal	73	47.4
Normal	81	52.6
SpO2		
Below 88.6	75	48.7
Between 88.6 and 100	79	51.3
Laboratory findings		
RBC count		
Below normal	16	10.4
Normal	138	89.6
WBC count		
Abnormal	14	9.1
Normal	140	90.9
Hemoglobin		
Below 9.1	24	15.6
Above 9.1	130	84.4
Platelets		
Abnormal	14	9.1
Normal	140	90.9
Cardiac biomarker		
Negative	41	26.6
Positive	113	73.4
Treatment		
Management		
Medical	153	99.4
Surgical	1	0.6
Final outcome		
Improved	98	63.6
Not improved	56	36.4
LOS in ICU		
Below 14 days	75	48.7
14 days or more	79	51.3

Risk factors associated with patients’ outcomes

By assessing the risk factors impacting patients’ outcome in terms of improvement after PE, patients’ demographics and medical history has no impact on the improvement of patients with PE (Table 3).

**Table 3:** Relationship between baseline characteristics and patients’ outcomes *Chi-square, **Monte Carlo Data has been represented as n and %. p-values less than 0.05 are considered statistically significant. %: percentage, n: number, PE: pulmonary embolism, DVT: deep vein thrombosis, CVA: cerebral vascular accident, DM: diabetes mellitus, HTN: hypertension, COVID-19: coronavirus disease 2019

Variables	Outcome (n (%))			p-value
	Improved (n=98) (63.6%)	Unimproved (n=56) (36.4%)	Total (n=154) (100%)	
Gender				
Female	48 (60.8)	31 (39.92)	79 (100)	0.446*
Male	50 (66.7)	25 (33.3)	75 (100)	
Smoking				
No	90 (65.7)	47 (34.3)	137 (100)	0.310**
Past smoker	1(50)	1 (50)	2 (100)	
Yes	7 (46.7)	8 (53.3)	15 (100)	
Pregnancy				
Male	50 (66.7)	25 (33.3)	75(100)	0.668*
Non-pregnant	45 (61.6)	28 (38.4)	73 (100)	
Pregnant	3 (50)	3 (50)	6 (100)	
Chronic disease history				
DM	9 (50)	9 (50)	18 (100)	0.850*
HTN	17 (63)	10 (37)	27 (100)	
HTN with DM	23 (63.9)	13 (36.1)	36 (100)	
Pulmonary disease with HTN or DM	3 (75)	1 (25)	4 (100)	
Others	9 (64.3)	5 (35.7)	14 (100)	
Free history	37 (67.3)	18 (32.7)	55 (100)	
Past medical history				
Cancer	7 (70)	3 (10)	10 (100)	0.372**
Cardiac disease	14 (82.4)	3 (17.6)	17 (100)	
Past COVID-19 infection	5 (41.7)	7 (58.3)	12 (100)	
CVA	7 (63.6)	4 (36.4)	11 (100)	
Hypothyroidism	4 (66.7)	2 (33.3)	6 (100)	
Pulmonary disease	2 (40)	3 (60)	5 (100)	
Others	16 (59.3)	11 (40.7)	35 (100)	
Free history	36 (62.1)	22 (37.9)	58 (100)	
History of DVT or PE				
DVT	12 (63.2)	7 (36.8)	19 (100	0.855*
PE	9 (60)	6 (40)	15 (100)	
Both of them	9 (75)	3 (25)	12 (100)	
None of them	68 (63)	40 (37)	108 (100)	
Prophylaxis by anticoagulant				
No	68 (63)	40 (37)	108 (100)	0.790*
Yes	30 (65.2)	16 (34.8)	46 (100)	
Previous surgery				
No	73 (66.4)	37 (33.6)	110 (100)	0.266*
Yes	25 (56.8)	19 (43.2)	44 (100)	
Surgery time				
Before 1-3 weeks	15 (55.6)	12 (44.4)	27 (100)	0.831*
Before 3-6 weeks	10 (58.8)	7 (42.2)	17 (100)	

Concerning the clinical characteristics of patients and their relation to patients’ outcomes (Table 4), the patients’ HR showed a statistically significant correlation with patients’ outcomes. Having an HR of less than 71.1 bpm had a significant impact on improving patients’ prognosis (p=0.003). No other factors showed any significant association with the patients’ outcomes (Table 4).

**Table 4:** Relationship between clinical characteristics and patients’ outcomes *Chi-square test, **Monte Carlo test Data has been represented as n and %. p-values less than 0.05 are considered statistically significant. %: percentage, n: number, PE: pulmonary embolism, HR: heart rate, SBP: systolic blood pressure, ABG: arterial blood gas, SpO2: saturation of peripheral oxygen, RBC: red blood cell, WBC: white blood cell, LOS: length of stay

Variables	Outcome (n (%))			p-value	
	Improved (n=98) (63.6%)	Not improved (n=56) (36.4%)	Total (n=154) (100%)		
Type of PE					
Massive	16 (61.5)	10 (38.5)	26 (100)	0.765*	
Non-massive	22 (59.5)	15 (40.5)	37 (100)		
Sub-massive	60 (65.9)	31 (34.1)	91 (100)		
Location of thrombus					
Pulmonary artery	4 (80)	1 (20)	5 (100)	0.058**	
Segmental	5 (100)	0 (0)	5 (100)		
Subsegmental	87 (63.5)	50 (36.5)	137 (100)		
Others	2 (28.6)	5 (71.4)	7 (100)		
Vital signs					
HR					
<71.1 bpm	14 (100)	0 (0)	14 (100)	0.003*	
71.1-105.7 bpm	57 (55.3)	46 (44.7)	103 (100)		
>105.7 bpm	25 (71.4)	10 (28.6)	35 (100)		
Blood pressure					
Above 130/73	47 (73.4)	17 (26.6)	64 (100)	0.100*	
Below 115/70	21 (55.3)	17 44.7)	38 (100)		
Between 115/70 and 130/73	30 (57.7)	22 (42.3)	52 (100)		
SBP					
Less than 90	16 (61.5)	10 (38.5)	26 (100)	0.807*	
More than 90	82 (64.1)	46 (35.9)	128 (100)		
Hemodynamic stability					
No	59 (64.1)	33 (35.9)	92 (100)	0.877*	
Yes	39 (62.9)	23 (37.1)	62 (100)		
ABG					
pH					
Abnormal	34 (58.6)	24 (41.4)	58 (100)	0.315*	
Normal	64 (66.7)	32 (33.3)	96 (100)		
CO2					
Abnormal	45 (61.6)	28 (38.4)	73 (100)	0.626*	
Normal	53 (65.4)	28 (34.6)	81 (100)		
SpO2					
Below 88.6	48 (64)	27 (36)	75 (100)	0.927*	
Above 88.6	50 (50)	29 (36.7)	79 (100)		
Laboratory findings					
RBC count					
Below normal	11 (68.8)	5 (31.3)	16 (100)	0.653*	
Normal	87 (63)	51 (37)	138 (100)		
WBC count					
Abnormal	10 (71.4)	4 (28.6)	14 (100)	0.523*	
Normal	88 (62.9)	52 (37.1)	140 (100)		
Hemoglobin					
Below 9.1	16 (66.7)	8 (33.3)	24 (100)	0.737*	
Above 9.1	82 (63.1)	48 (36.9)	130 (100)		
Platelet count					
Abnormal	10 (71.4)	4 (28.6)	14 (100)	0.525*	
Normal	88 (62.9)	52 (37.1)	140 (100)		
LOS					
Below 14 days	47 (62.7)	28 (37.3)	75 (100)		0.807*
14 days or more	51 (64.6)	28 (35.4)	79 (100)		

Correlation between patients’ outcomes and age

By applying binary logistic regression, the age had no statistically significant relation with the patients’ outcomes in terms of improvement after PE (Table 5).

**Table 5:** Binary logistic regression between patients’ outcomes and age Data has been represented as odds ratio and 95% CI. p-values less than 0.05 are considered statistically significant. CI: confidence interval

Variables	B coefficient	Odds ratio	95% CI		p-value
			Lower	Upper	
Age	0.004	1.004	0.987	1.021	0.656

## Discussion

This study shed light on the risk factors that impacted PE prognosis in terms of improvement after PE. Decreased HR has a significant impact on improving the prognosis in all patients.

In the current study, most of the native high-altitude PE patients had an improvement after having PE (63.6%, n=98). Few cases were reported of PE among patients at high altitudes [10,11]. To the best of our knowledge, no prior studies investigated patient outcomes after PE among patients at high altitudes and the prognostic factors associated with these outcomes.

Regarding the impact of low HR, in accordance with our findings, Jaureguízar et al. [12] conducted a study on the association between HR and PE. They found that low HR was associated with low mortality and a borderline significant correlation between increased HR and in-hospital death. However, in our study, slightly higher HR had a positive prognosis, which is in contrast to several previous findings [13]. This could be because the blockage causes pulmonary damage and reduces the oxygen amount in the blood. As a result, a slightly increased heart rate compensates for the blockage, resulting in a better prognosis.

In terms of thrombi location, García-Sanz et al. [14] and Cha et al. [15] reported that patients with subsegmental PE had a higher survival rate. However, our study concluded that the thrombus location had no significant correlation with PE patients’ outcomes.

Regarding age as a predictor of PE prognosis, Cefalo et al. [16] and Spirk et al. [17] reported higher mortality rates among massive PE patients aged 65 years or older. However, in the current study, age was not found to affect PE prognosis. This could be explained by the small percentage of massive PE in our study.

Additionally, Secemsky et al. [18] and Meneveau et al. [19] found that massive PE patients had greater mortality rates compared with sub-massive PE patients. Rehman et al. [20] reported that thrombolysis is associated with improved outcomes for sub-massive PE, with no significant effect on mortality or major bleeding. Several studies estimated that the 90-day mortality rates were 58.3% in patients with massive PE versus 15.1% in those with sub-massive PE and 2% in patients with low-risk PE [21]. Our study found no significant results with PE patients’ outcomes. In our study, a low percentage of the included patients had massive PE, which could justify our findings.

Furthermore, Quezada et al. [22] and Keller et al. [23] reported that systolic blood pressure < 100 mmHg causes an adverse outcome among acute PE patients. However, our results concluded no significant results with PE patients’ outcomes. This could be explained by the small percentage of SBP < 90 mmHg in our study from all included PE patients (16.9%, n=26). Moreover, according to Keller et al. [23], systolic and diastolic BPs are excellent prognosis predictors of patients with acute PE. SBP of 120 mmHg or less and diastolic blood pressure (DBP) of 65 mmHg or less at admission are connected with elevated risk of in-hospital death.

Dentali et al. [24] and Smith et al. [25] reported a significant reduction in PE incidence during anticoagulant prophylaxis, reduced mortality in patients with acute PE, and decreased recurrence.

PE is a serious and common complication of several surgery types, especially general surgery [8]. In addition, PE development could be highest within the first five weeks after surgery [7]. In the present study, about one-third of the patients underwent a previous surgery within 1-6 weeks. However, neither undergoing surgery nor surgery time significantly affected the outcome among patients. On the contrary, a prior study found that patients who had recent surgery had higher 30-day mortality [25].

Limitations

Due to the retrospective nature of the study design and the small number of patients it included, the validity of our findings is limited. Future prospective study involving a larger sample size is required to verify our findings.

## Conclusions

This study provides insights into the outcomes of PE at high altitudes and the factors influencing these outcomes after PE. Moreover, the study contributes to the understanding of risk factors associated with PE prognosis in terms of patient improvement. Based on the severity of the PE, the study did not find any appreciable variations in patient outcomes. Low HR was found to have a beneficial effect on PE improvement. Therefore, attention should be given to the impact of PE patients’ HR on their recovery.
